# Arbuscular Mycorrhizal Fungi from Argentinean Highland Puna Soils Unveiled by Propagule Multiplication

**DOI:** 10.3390/plants10091803

**Published:** 2021-08-30

**Authors:** Fernanda Covacevich, Keren Hernández Guijarro, Esteban M. Crespo, Erica Lumini, María Soledad Rivero Mega, Mónica A. Lugo

**Affiliations:** 1 Instituto de Investigaciones en Biodiversidad y Biotecnología (INBIOTEC-CONICET) Fundación para las Investigaciones Biológicas Aplicadas (FIBA), Mar del Plata 7600, Argentina; 2 Instituto Nacional de Tecnología Agropecuaria (INTA), Unidad Integrada Estación Experimental Agropecuaria Balcarce, Balcarce 7620, Argentina; hernandez.keren@inta.gob.ar; 3 Micología, Diversidad e Interacciones Fúngicas (MICODIF), Área Ecología, Facultad de Química, Bioquímica y Farmacia, Universidad Nacional de San Luis (UNSL), San Luis 5700, Argentina; ecrespo@unsl.edu.ar (E.M.C.); mariasoledadrivero@sanluis.edu.ar (M.S.R.M.); lugo@unsl.edu.ar (M.A.L.); 4 Institute for Sustainable Plant Protection (IPSP) of National Research Council (CNR), Viale Mattioli 25, 10125 Turin, Italy; 5 Instituto Multidisciplinario de Investigaciones Biológicas (IMIBIO-CONICET-UNSL), San Luis 5700, Argentina

**Keywords:** Glomerales, trap plant multiplication strategy, biodiversity, highlands, single strand conformation polymorphism (SSCP)

## Abstract

Low arbuscular-mycorrhizal (AM) sporulation in arid field soils limits our knowledge of indigenous species when diversity studies are based only on spore morphology. Our aim was to use different approaches (i.e., spore morphological approach and PCR–SSCP (single-strand-conformation-polymorphism) analysis after trap plant multiplication strategies to improve the knowledge of the current richness of glomalean AM fungi (Glomerales; Glomeromycota) from the Argentine Puna. Indigenous propagules from two pristine sites at 3870 and 3370 m of elevation were multiplied using different host plants; propagation periods (2–6 months), and subculture cycles (1; 2; or 3) from 5 to 13 months. The propagule multiplication experiment allowed the detection of different glomoid taxa of *Funneliformis* spp. and *Rhizoglomus* spp., which were considered cryptic species since they had never been found in Puna soils before. On the other hand; almost all the generalist species previously described were recovered from cultures; except for *Glomus ambisporum*. Both plant host selection and culture times are critical for Glomerales multiplication. The SSCP analysis complemented the morphological approach and showed a high variability of *Glomus* at each site; revealing the presence of *Funneliformis mosseae*. This study demonstrates that AMF trap culture (TC) is a useful strategy for improving the analysis of AM fungal diversity/richness in the Argentinean highlands.

## 1. Introduction

Arbuscular mycorrhizal fungi (AMF) favour plant growth by playing an important role in the exchange of nutrients and metabolites. Diversity studies of AMF in this ancient symbiotic relationship are carried out through both classical taxonomy and molecular techniques provide a better understanding of their different functions and roles in ecosystem functioning [1]. 

AMF diversity has been traditionally assessed by the morphological identification of fungal spores [2]. Nevertheless this methodology poses some difficulties: (i) spore production is highly dependent on AMF physiology and the environment (edaphic and climatic); (ii) some fungi are non-sporulating; and (iii) spores could be parasitized or degraded in the soil [3]. Despite its detractors, AMF trap culture (TC) has been successfully implemented to circumvent these problems and to multiply soil AMF propagules under controlled conditions to increase the chances of species detection [3,4] and possibly serve as a source of an accurately identified AMF inoculum. Besides the morphological characterisation of AMF spores, a plethora of molecular techniques has been usefully developed in the last decades that allow the assessment of both AM fungal diversity from different ecosystems (i.e., forest, grassland, rangelands, and agroecosystems) [5] and the genetic diversity of specific AM fungal taxa (i.e., different karyotypes produced by the genus *Funneliformis* (= *Glomus*) and the wide interisolate genetic diversity of model AMF such as *Rhizophagus irregularis*) [6,7]. Next-generation sequencing (NGS) allows for better characterization of AMF communities in different ecosystems. However, its cost and the computational capacity needed to process and understand the huge amount of information generated limit the extensive adoption of these techniques. On the other hand, fingerprinting analysis based on DNA banding patterns has been broadly used for decades to unveil the genetic variability of soil microbial communities (i.e., denaturing and temperature gradient gel electrophoresis (DGGE and TGGE, respectively), single-strand conformation polymorphism (SSCP), length heterogeneity-PCR (LH-PCR), terminal-restriction fragment length polymorphism (T-RFLP)). At present, these techniques are still valid due to their high genotypic resolution, low cost and high versatility, reliability and reproducibility. Among these techniques, SSCP has been widely used for both assessing AMF diversity in peculiar environments, such as arid gypsum sites [8], and detecting shifts in soil fungal community structure related to different land uses and agricultural management [9,10]. Thus, the use of SSCP could be considered a first approach before deciding to perform NGS.

Despite the growing interest in knowing and safeguarding the biological diversity associated with extreme environments, some biodiversity hotspots such as the Argentine Puna are being increasingly threatened and not extensively investigated. The Puna is a harsh South American biogeographical region characterised by highlands with a desert climate and unique properties, such as a wide daily temperature range, high solar radiation, and particular flora and fauna [11]. AMF diversity has been scarcely studied in this environment [12]. Different AMF indigenous species from field soils at elevations higher than 3320 m have been identified by means of direct spore isolation and their morphological characterisation [12] without previous propagule multiplication. A low number of AMF spores directly retrieved from field samples and an inverse relationship between AMF spore diversity and height above sea level were found in Puna [12].

This work aims to evaluate the impact of soil propagule multiplication on the diversity and richness of the indigenous glomoid AMF species detectable in two soils of the Puna by comparing the results with those previously obtained, without multiplication, on field soils of the same sites: Abra del Cóndor (AC) and Iturbe (It). We hypothesised that different strategies for soil AMF propagule detection (direct propagule isolation vs TC multiplication) and characterisation (morphological and molecular SSCP approaches) could improve knowledge about the AMF community composition in this extreme environment. 

## 2. Results and Discussion

### 2.1. Glomoid AMF Diversity Assessed by Spore Morphology

**Figure 1:**
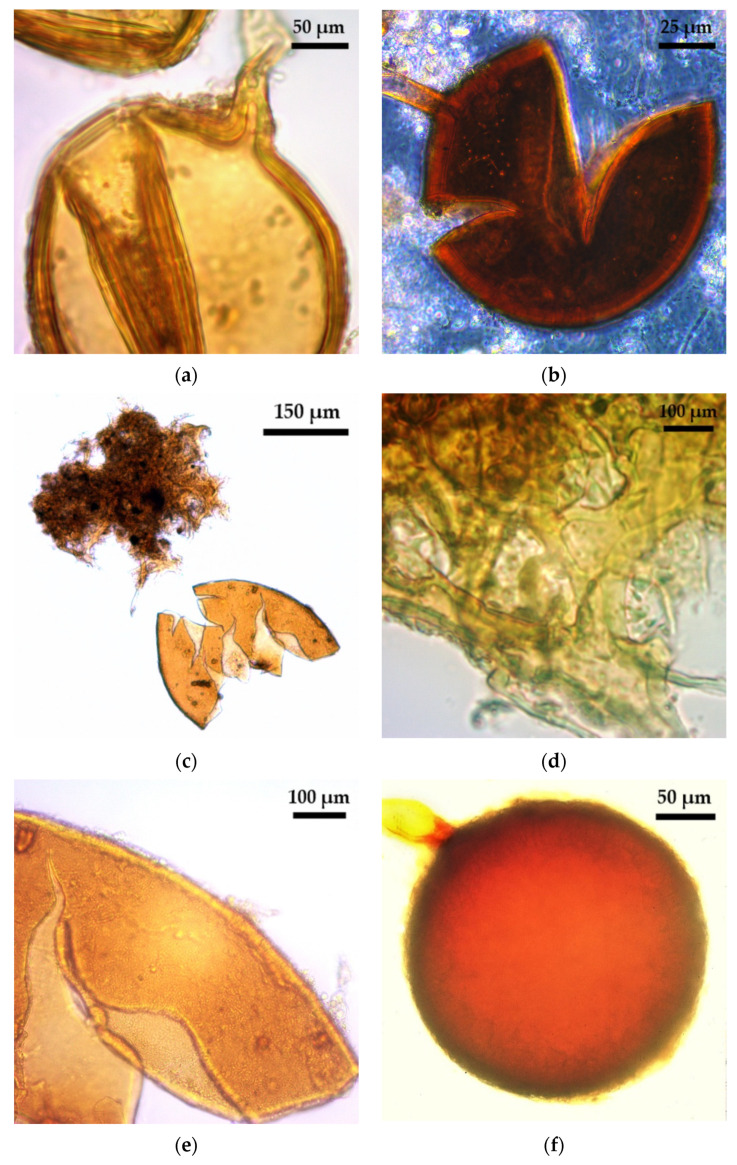
Glomoid AMF with single spores. (**a**) *Funneliformis* sp.; (**b**) *F. geosporus*; (**c**) *F. monosporus*, general view of the spore and hyphal mantle or peridium; (**d**) *F. monosporus*, detail of the hyphal peridium; (**e**) *F. monosporus*, detail of wall ornamentations; (**f**) *Septoglomus constrictum*.

**Figure 2:**
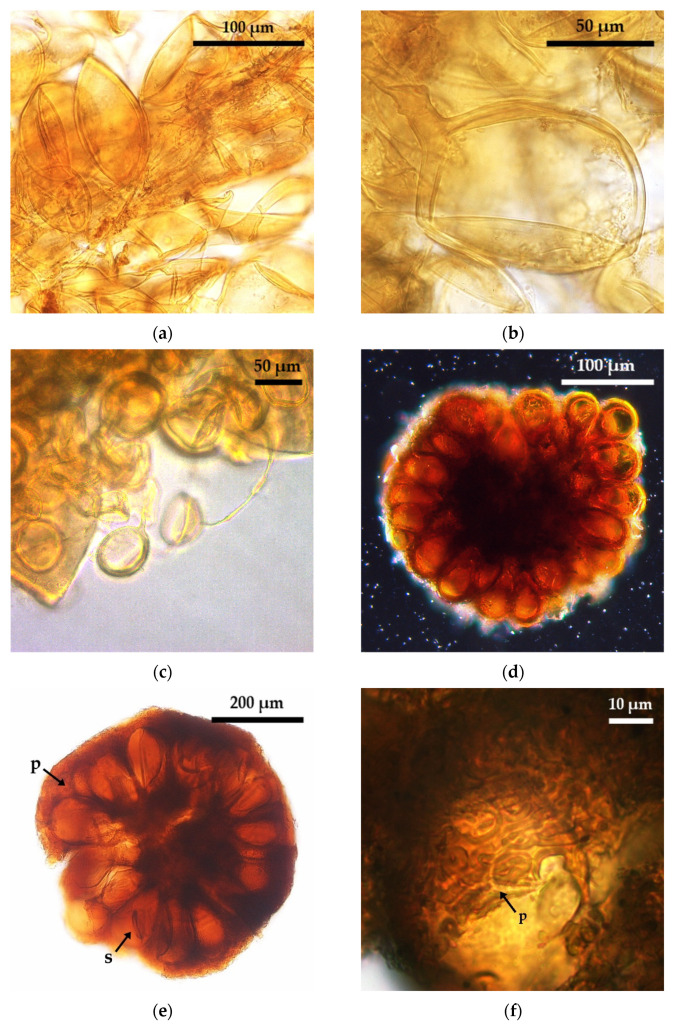
Glomoid AMF with spores in clusters and sporocarps. (**a**) *Rhizoglomus aggregatus* spore cluster in senescent root; (**b**) *R. aggregatus* spore with endospore; (**c**) *R. microaggregatum* spores inside other AMF spore; (**d**) *Sclerocystis* sp., general view of the spores without peridium; (**e**) *S. sinuosa*, general view of the spores (s) with peridium (p); (**f**) *S. sinuosa*, detail of the hyphal peridium (p).

Spores and sporocarps of glomoid arbuscular mycorrhizal fungi (AMF) were found in all the analysed samples (Figure 1, Table 1, Figure 2, Table 2). Different levels of morphospecies richness of glomoid spores, both with and without funnel shaped pores, were found at each trap culture (TC) multiplication cycle. Glomoid spores, derived from trap substrates (Table 1 and Table 2) were found in the form of single spores (Figure 1), in clusters and forming sporocarps, and within old roots and other AMF spores (Figure 2). Eight morphologically distinctive AMF species were retrieved (Table 1 and Table 2). All the analysed morphospecies from the Puna soils showed morphometric values lower than those reported for the original descriptions. Spore clusters of *Funneliformis* sp. (Figure 1a) and *F. monosporus* (Figure 1c–d) showed morphological and morphometric features similar to *Funneliformis mosseae,* but the last spore wall did not stain with a Melzer reagent.

The morphospecies *Funneliformis geosporus* (Figure 1b) was the most frequently found in all TCs, regardless of site (AC or It) or the analysed trap plant species (TPS) combination (Figure S1). However, this morphospecies was not found in field soils from AC (Table 1) or It (Table 2). Generally, *F. geosporus* is a quickly sporulating species but its spore abundance, both from field soil and TC, varies depending on land use [13] and edaphic properties [14]. In general, some authors suggest that AMF yield spores after 10 weeks of TC multiplication, which is in accordance with the previous two months’ report for *F. geosporus* TC [13,15]. This study showed that *F. geosporus* produced spores under the aforementioned multiplication conditions, which reinforces the importance of TC for spore production and the identification of this species in Puna soils. Therefore, *F. geosporus* was a cryptic AM species concerning Puna field samples but a generalist species in TC as it has been reported after different TC approaches were used in several environments [4,14,15,16]. In this study, trap culture allowed the development and sporulation of AMF species which otherwise could not have been recovered from field soil. 

*Septoglomus constrictum* (Figure 1f) is another cryptic species recovered only from Abra del Cóndor (AC) soils by means of TC with the C_4_ grass *Sorghum bicolor* as TPS in at least one culture cycle (Table 1). *S. constrictum* has also been reported as a cryptic species for different soils and environments [4] using 2 C_4_ TPS combined with other TPS in multiplication strategies with long-term culture periods in Brazilian tropical soils [4] and Chinese saline and forest soils [15]. However, *S. constrictum* could not be recovered in an 8-month TC with 4 different TPS that were not C_4_ [13]. Based on this evidence and on the fact that AMF sporulation is a host species-dependent process [4], the results suggested that the inclusion of a C_4_ TPS in the TC could be necessary to promote the sporulation of *S. constrictum* from AC soils. As regards Iturbe (It) soil (Table 2), *S. constrictum* could not be detected, even when C_4_ species were used as a TPS. Further investigation should be carried out to determine whether this species recovery is influenced by other factors.

The aggregate-forming morphospecies *Rhizoglomus aggregatus* (Figure 2a,b) and *R. microaggregatum* (Figure 2c) were detected only in It samples by means of TC. *R. microaggregatum* was not detected either in Abra del Cóndor TC or in Iturbe field samples [12], which suggests that *R. microaggregatum* is another cryptic species revealed by TC. However, taking into account the different results obtained with the three TC strategies used for the It samples (no *R. microaggregatum* at the soil from ItM3-a2, see Table 2), it can be inferred that subsequent cultivation cycles with *S. bicolor* could inhibit *R. microaggregatum* sporulation. Furthermore, other studies showed that *R. microaggregatum* can be found in field sample soils, but that it cannot be recovered after 5 months of TC that included a single TPS per pot [14]. We therefore suggest that to achieve successful sporulation of *R. microaggegatum*, a TC strategy should include more than one TPS per TC cycle; several cycles of more than 5 months of cultivation, alternating TPS between cycles; and, in a third cycle, a TPS different from *S. bicolor*.

On the other hand, *R. aggregatus* (Figure 2a,b) was previously detected in It and AC soil samples [12], but it was not recovered from AC soils by TC in spite of being considered both a cryptic species for tropical areas [4] and a generalist species for field and meadow European soils [13] and Chinese mountains [15]. In these studies, *R. aggregatus* sporulation in TC was strongly influenced by TPS [14] and it only required an average of 4 months of multiplication [13,15]. Our results also reflect this trend in which the use of numerous TPS and a long cultivation period in TC (at least 13 months) promoted the sporulation of *R. aggregatus*.

Regarding *Glomus ambisporum*, it was detected in field soils [12], but it could not be recovered with TC multiplication, which was in agreement with previous reports where it was regarded as a common species in field soils but an absent one in TC along diverse environments and trap strategies [4,13,15]. Future studies with in vitro culture or direct detection by molecular tools are necessary to detect and isolate *G. ambisporum*, which has shown to be difficult to culture with the TC methodology.

Two sporocarpic morphospecies of *Sclerocystis, Sclerocystis* sp. (Figure 2d) and *S.* s*inuosa* (Figure 2e,f), were found at the two sites after TC. Nevertheless, *S. sinuosa* was not detected in any soil sample whenever *A. ampeloprasum* var. *porrum* was included as a TPS. This result deserves future study as *S. sinuosa* is a generalist AMF in Puna field soils [12]. Moreover, other studies show that, even though *S. sinuosa* is commonly found in field samples from tropical and Alpine environments [13,14], it could not be recovered by TC. Regardless, the application of TC strategies was successful in promoting *S. sinuosa* sporulation in Puna soils when the rest of the TPS were used.

### 2.2. Molecular Analysis of Glomoid AMF Diversity

**Figure 3:**
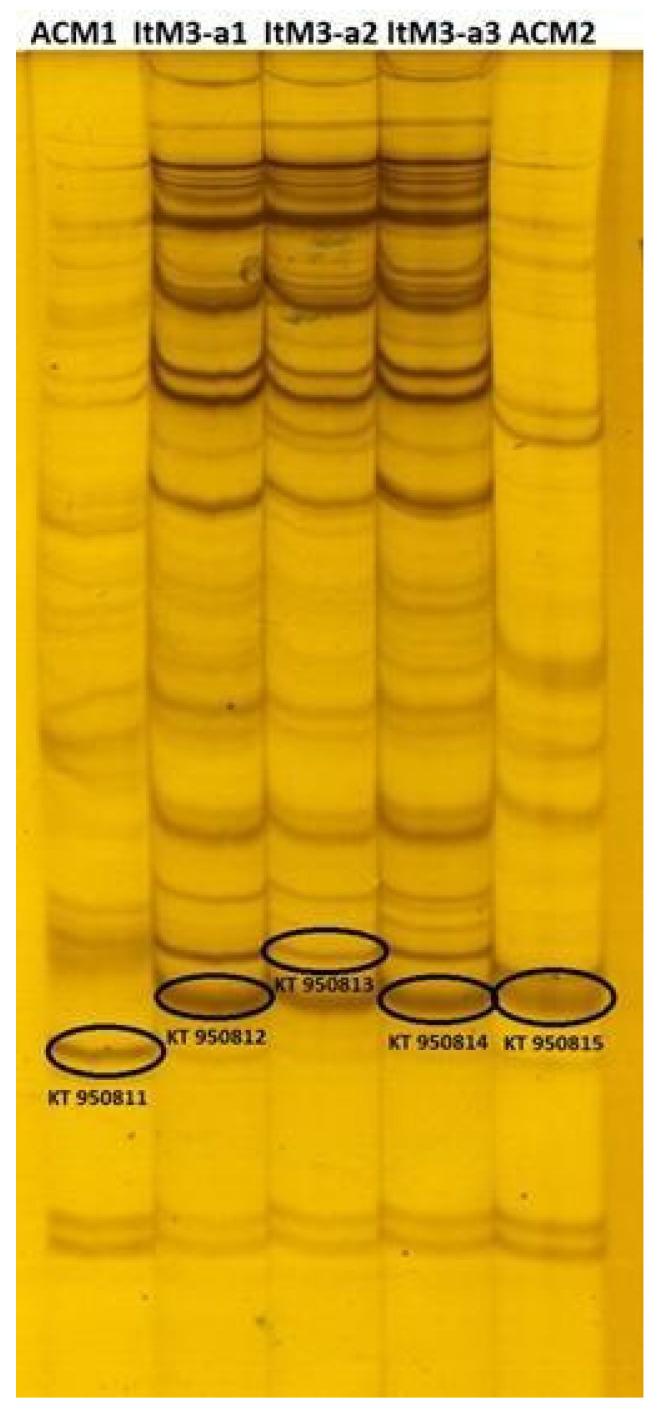
PCR–SSCP patterns obtained from a region of the 28S-rDNA gene of AMF indigenous to the Argentine Puna belonging to *Funneliformis* genus. References: AC: Abra del Cóndor; It: Iturbe; M1, M2, M3: multiplication cycles 1, 2 and 3, respectively. Bands marked with ovals were cut and sequenced. Access numbers of the sequences are shown on Figure 4.

The diversity of glomoid arbuscular mycorrhizal fungi (AMF) indigenous to the Puna soils was assessed through the polymorphism of the 28S rDNA gene using a specific set of primers that amplified representative species of *Funneliformis* (= *Glomus*) genus such as *F. mosseae*, *F. caledonius,* and *F. geosporus* [16,17]. The single-strand conformation polymorphism (SSCP) assay revealed the genetic variability of the glomoid AMF in soils from Abra del Cóndor (AC) and Iturbe (It). The number of detected bands ranged between 16 and 38 for all samples. Some band patterns were common to all sites and multiplication strategies, while others were site-specific (Figure 3).

Richness, which is the number of taxa (phylotype number) per sample, was determined from SSCP fingerprints. For Abra del Cóndor soil, we found 16 phylotypes (distinctive bands) at the ACM2 TC strategy and 29 at the ACM1 (for TC details see Figure S1, Table 1). For the Iturbe soils, we found 32, 36 and 38 phylotypes for strategies ItM3-a1, ItM3-a2 and ItM3-a3, respectively (Figure S1, Table 2). Differences in richness were also reflected in the Shannon–Wiener H’ index, which was calculated from the number and intensity of the bands from the pattern generated by the SSCP strategy (Figure 3). Among the soils of Abra del Cóndor, the ACM1 strategy seemed to increase H’ diversity, while for the Iturbe soil the H’ index was similar among all TC strategies. 

The richness obtained from the SSCP strategy was higher than the one obtained from the spore morphology (Table 1 and Table 2). The morphological analysis focused on the taxonomic determination of entire spores, while the SSCP strategy started with DNA extraction from soil where all genomic material could be extracted from both spores and other AMF structures (hyphae) in the soil. In this sense, even though it seemed that this could be the cause of the higher richness revealed by the SSCP approach in comparison to the morphological one, in a previous study [9] we determined that both the diversity and richness of AMF evaluated by the SSCP strategy did not differ between analysis from soil (spores + hyphae) or from previously isolated AMF spores. We did not know the causes of the lower richness revealed by the morphological approach; hence, future studies should focus on elucidating this matter. 

**Figure 4:**
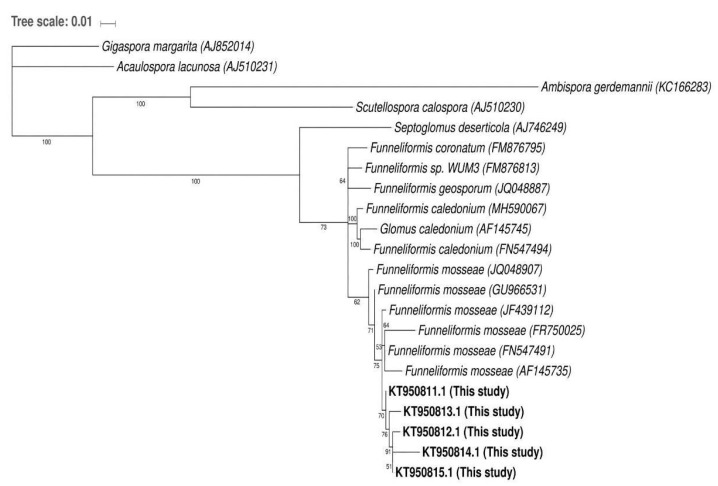
Phylogenetic tree constructed with a maximum parsimony analysis of taxa with the obtained SSCP sequences of closely related AMF species. References: the branches corresponding to partitions reproduced in less than 50% of bootstrap replicates are collapsed. The percentage of replicate trees in which the associated taxa clustered together in the bootstrap test (100 replicates) is shown next to the branches. Sequences generated in this study were registered in GenBank under the following accession number: KT950811, KT950812, KT950813, KT950814 and KT950815.

The sequences obtained from the re-amplified bands of the polyacrylamide gel showed considerable similarity (>97%) with the representative sequences of *Funneliformis* (= *Glomus*) genus available in the databases. This genus was grouped separately from other AM fungal genera and was included as an outgroup (Figure 4). The SSCP revealed the presence of *Funneliformis mosseae* although this species was not detected by the morphological spore analysis. This result reinforced the importance of complementing classical morphological techniques with molecular ones such as the SSCP strategy for prospective AMF diversity studies.

Although the morphological analysis allowed a more comprehensive differentiation within an AM fungal genus than the molecular analysis, some difficulties related to the recognition of the juvenile, mature, and old spores of each species were frequently reported [18]. Regarding TC, only a low percentage of studies recommended it as a reliable method for AMF multiplication prior to morphological or molecular identification. Some authors argue that TC does not reflect in situ reality [19]; others [20] state that TC should not be used to assess AMF diversity whenever the richness is less than 21 species per site, whereas the sole use of direct analysis of field samples should be avoided if the total AMF richness is below that value. However, it was demonstrated [21] both by morphological and molecular methods that a considerable proportion of spores in the rhizosphere were dead despite their intact appearance. The authors then stated that using TC would give more reliable results for AMF diversity studies. Furthermore, it was also demonstrated that the denaturing gradient gel electrophoresis (DGGE) clustering analysis was reliable for the detection of AMF diversity after TC and that AMF diversity increased more significantly when combined with three host species (*Trifolium repens*, *S. bicolor*, and *Zea mays*) than with one host species grown alone [22]. DGGE is a strategy comparable to SSCP; however, the latter is sometimes more efficient at detecting different phylogenetic groups and less time-consuming for sample preparation [23]. Furthermore, the SSCP approach revealed the presence of *F. mosseae*, a glomoid species that remained hidden for the morphological methodology. This result is in accordance with new molecular results obtained by the next-generation sequencing approach applied to Puna soils of native *Z. mays* var. *ocho-rayas* and *Solanum tuberosum* var. *chusqueña* at Chaupi Rodeo, an Andean settlement located near the sites studied in this work [24].

## 3. Materials and Methods

### 3.1. Study Area and Soil Sampling

The study area is located in the floristic district known as “Puna Seca”, Argentina. Two pristine sites were analysed: Abra del Cóndor (AC) (22°53′23.5″ S; 65°14′56.1″ W, 3870 m a.s.l., Salta Province) and Iturbe (It) (23°00′06.8″ S; 65°22′07.31″ W, 3370 m a.s.l., Jujuy Province; Argentin). Thirty sub-samples of soil and roots from native grasses were collected at each site from an area of 1 ha at a depth of 15–20 cm. Only plants of the grass family Poaceae were sampled to minimize the effect of the host species on the AMF communities. The climatic characteristics, soil properties, and grass species [12,25,26] of the sampled sites are detailed in the supplementary information (Tables S1–S3).

### 3.2. AMF Propagule Multiplication

The collected soils were used as substrates to perform the trap cultures (TCs) using different trap plant species (TPS) as arbuscular mycorrhizal fungi (AMF) hosts (Table 1 and Table 2, Figure S1). All seeds of the TPS were surface sterilized by immersion in 90% ethanol for 3 min and in 5% NaClO for 1 min. The TPS were grown in pots of 2 kg under controlled conditions: 12 h light/12 h dark, 21 °C, and at 60% of the soil water-holding capacity throughout the experiment. Each TC multiplication cycle was quintupled. At the end of each cycle, and regardless of the number of TPS used, all substrates were homogenised and used for subsequent analyses (Figure S1). 

Soil samples from Abra del Cóndor (AC) were grouped into ACM1 and ACM2 according to the TC strategies, which differed both for the diversity of the TPS and the number and duration of the multiplication cycles. ACM1 and ACM2 included 1 and 2 culture cycles of 5 and 7 months, respectively (Table 1, Figure S1). Soil samples from Iturbe (It) were divided according to the treatments into ItM3-a1, It M3-a2, and It M3-a3 (Figure S1). The multiplication periods and the number of culture cycles were common to all treatments: 13 months divided into 3 cycles of 5, 6, and 2 months. The first and second cycles included the same TPS for all treatments. The third treatment used a different TPS for each treatment (Table 2, Figure S1).

### 3.3. Glomoid AMF Diversity Assessed by Spore Morphological Approach

AMF spores were extracted from TC substrates using the wet sieving method, for which they were successively passed through 250, 125, and 38 μm sieves, and then centrifuged in a 50% sucrose solution [2]. Only glomoid spores were morphologically studied since this was the only AMF group that could also be analysed with molecular tools. Spores were mounted onto slides using PVLG (polyvinyl alcohol-lactic acid-glycerol) both with and without a Melzer reagent. The taxonomic classification of Glomeromycota was carried out by microscopic observation of the spores, which were compared with the descriptions available in http://www.amf-phylogeny.com/, http://fungi.invam.wvu.edu/the-fungi/species-descriptions.html (accessed on 7 July 2021) and [27]. Voucher specimens of spores and sporocarps were deposited in MICODIF-Unit 266–360-SNDB-UNSL (Sistema Nacional de Datos Biológicos-Universidad Nacional de San Luis, Argentina).

### 3.4. Molecular Analysis of Glomoid AMF Diversity

AMF genetic diversity was analysed with PCR-single strand conformation polymorphism (PCR–SSCP). The DNA was isolated from 0.25 g of each trap culture substrate (homogenised from replicates) obtained after the treatments previously described using the PowerSoil^®^ DNA Extraction kit (MoBio, Germantown, MD USA) according to the manufacturer’s instructions. Afterwards, nested PCRs were performed to amplify a fragment of the large subunit of the 28S rRNA gene. PCR amplifications were run using an Eppendorf Thermal Cycler (Bio-Rad, Hercules, CA, USA). For the first reaction, 3 µL of template (extracted DNA) were amplified by using 1 U of the GoTaq^®^ DNA Polymerase (Promega), its buffer, needed dNTPs, and the fungal specific primer pair LSU0061/LSU0599 [16] (5′-AGCATATCAATAAGCGGAGGA-3′/5′-TGGTCCGTGTTTCAAGACG-3′). The following program was used: an initial denaturation for 2 min at 94 °C was followed by 30 cycles of 1 min at 94 °C, 1 min at 53 °C and 5 min at 72 °C, and 7 min at 72 °C. The second reaction was performed with 3 μL of template (1:50 dilution of amplicons resulting from the first PCR reaction), 1 U of the GoTaq^®^ DNA Polymerase (Promega), its buffer, the corresponding dNTPs, and AMF specific primer pair LSUrk4f/LSUrk7r [16,17] (5′-GGGAGGTAAATTTCTCCTAAGGC-3′/5′-ATCGAAGCTACATTCCTCC-3′). Nested PCR program was as follows: an initial denaturation of 2 min at 94 °C, 25 cycles (1 min at 94 °C, 1 min at 60 °C, and 1 min at 72 °C), and a final extension (7 min at 72 °C). This primer pair was suitable for the SSCP analysis and targeted a sequence of 300 bp, including the LSUrDNA variable domain D1 from a broad spectrum of AMF species that belong to *Funneliformis* spp. (i.e., *Funneliformis mosseae*; *F. caledonius* and *F. geosporus*) and *Rhizoglomus*. Integrity and concentration of extracted DNA and size of PCR amplicons were checked by agarose gel electrophoresis (1.0% w/v agarose; 100 V, 45 min) using Gel Red^®^ staining.

About 5 µL of the PCR products generated by LSUrk4f and LSUrk7r primer pairs were analysed by the SSCP technique. The amplicons were denatured with 3 µL of denaturing loading mixture (95% deionized formamide, 0.05% bromophenol blue, 0.05% xylene cyanol FF and EDTA 20 mM) at 95 °C for 5 min, and immediately plunged into ice. Each sample was loaded into the 1 mm-thick gel 0.5X MDE^®^ (Cambrex, Rockland ME, USA), the run buffer consisted in 1X TBE (0.045 M Tris/borate, 0.001 M EDTA). Electrophoresis was performed at e Dcode Universal Mutation Detection System (Bio-Rad, USA) at 15 °C, 8 W, 300 V for 4 h. The SSCP gels were silver stained following [28], scanned, and finally analysed. The protocols were performed as in previous studies [9,16]. The SSCP patterns (presence/absence and intensity of bands) were analysed using the Phoretix1DPRO software (Nonlinear Dynamics, United Kingdom). Only bands with an intensity >0.1% of the total lane were considered for further analysis. All richness and diversity indices were calculated using the 0.1% threshold for relative abundances of single phylotypes (bands) because without such a detection limit these indices are not reliable [29]. Number and band intensity were used to calculate the Shannon–Wiener diversity H’ index [30] and AMF richness was indicated by the total number of bands detected.

One defined band per sample from the SSCP gel was excised, suspended in 30 µL water (2 cycles of hot–freeze: 1 h at 60 °C and frozen at −20 °C), re-amplified (as described for second PCR reaction), purified (Wizard^®^ SV Gel and PCR Clean-Up System), and sequenced (Macrogen). Homologous sequences to those obtained in this study were determined through BLASTn procedure at NCBI and MAARJAM (http://maarjam.botany.ut.ee, accessed on 7 July 2021) databases. Phylogenetic analysis was carried out using *Funneliformis* sequences which were selected following the criteria of query coverage between 99 and 100%, Maximun identity >97%, and the E values > 1 × 10^−100^ (Table S4). Sequences from well-defined organisms were selected instead of sequences from unculturable organisms to develop a better understanding of sequence-based species identification of the Glomeromycota genus *Funneliformis* (= *Glomus*). Sequences that belonged to other Glomeromycota genera such as *Septoglomus*, *Scutellospora*, *Ambispora*, *Acaulospora*, and *Gigaspora* were included in the analysis as outgroups. All sequences were aligned using T-Coffee software (multiple sequence alignment: MSA), and a maximum-likelihood tree was performed using the PhyML 3.0. platform (HKY85 substitution model, SPR algorithm, 100 bootstraps) [31]. All sequences obtained in this study were deposited at NCBI Gene Bank under the accession numbers KT950811 (ACM1), KT950812 (ItM3-a1), KT950813 (ItM3-a2), KT950814 (ItM3-a3), and KT950815 (ACM2).

## 4. Conclusions

In this study, different strategies of multiplication of arbuscular mycorrhizal fungi (AMF) propagule indigenous to two pristine soils of the Argentine Puna (elevation over 3000 m a.s.l.) were evaluated. The diversity and richness of species with an emphasis on glomoid AMF were analysed, using both a morphological approach as well as a simple and low-cost molecular tool. Our results were compared with previous field AMF spore diversity reports at these sites, which confirmed the effectiveness of TC in detecting cryptic AMF species never before described for the Puna. Seven cryptic AMF species were identified by spore morphology (*Funneliformis* sp., *F. geosporus*, *F. monosporus*, *Rhizoglomus microaggregatum*, *Septoglomus constrictum,* and *Sclerocystis* sp.) and by sequencing (*F. mosseae*). The generalist AMF identified included *Funneliformis* sp., *F. geosporus*, *F. mosseae*, *R. microaggregatum*, *R. aggregatus*, *Sclerocystis sinuosa,* and *Septoglomus constrictum*, while some rare species included *F. monosporus* and *Sclerocystis* sp. The combination of TPS used was crucial to detecting cryptic glomoid species. The SSCP in combination with the morphological approach, allowed to characterise the diversity of the indigenous glomoid AMF multiplied in TC from the studied sites. The results of this study seem to indicate that the TC approach played an important role in multiplying AMF propagules, and that increasing the number of multiplication cycles did not negatively impact the composition and richness of the glomoid AMF. Overall, we emphasized the importance of combining morphological and molecular strategies in diversity studies and the maintenance of AMF diversity through the variation of host plants and the application of long multiplication cycles for propagules multiplication.

## Figures and Tables

**Table 1:** Description of trap plant culture cycles, glomoid species found and richness assessed at the Puna site Abra del Cóndor.

ABRA DEL CÓNDOR			
Trap Culture Strategy	AC Field	ACM1	ACM2
Sampling site of Puna	Abra del Cóndor	Abra del Cóndor	Abra del Cóndor
Site elevation	3870 m	3870 m	3870 m
Data source	[12]	This study	This study
Initial total spore density/100 g dry soil weight	-	424	424
1st cycle time	-	5 months	5 months
1st cycle TPS	-	* Sorghum bicolor * + *Melilotus albus*	* S. bicolor *
2nd cycle time	-	-	2 months
2nd cycle TPS	-	-	* S. bicolor +M. albus + * * Allium ampeloprasum * var. *porrum*
Total time of trap culture	-	5 months	7 months
Glomoid AMF taxa	* Glomus * sp. *G. ambisporum* *Rhizoglomus aggregatus* (=*Glomus aggregatum*) *Sclerocystis sinuosa* (=*Glomus sinuosum*)	* Funneliformis * sp. *F*. *geosporus* *Sclerocystis* sp. *S. sinuosa* *Septoglomus constrictum*	* Funneliformis * sp. *F. geosporus* *Septoglomus constrictum*
Richness of glomoid morphospecies with funnel pore	0	3	3
Richness of total glomoid morphospecies	4	5	4
H’ Diversity Index (SSCP analysis)	-	1.44	1.16
Sequence of an excised band (SSCP analysis)	-	KT950811	KT950815

AC: Abra del Cóndor; AC Field: Abra del Cóndor soil field samples; ACM1: Abra del Cóndor soil samples with one cycle of trap culture; ACM2: Abra del Cóndor soil samples with two cycles of trap culture; SSCP: single-strand conformation polymorphism; TPS: trap plant species; -: no trap culture was carried out.

**Table 2:** Description of cycles of trap plant cultures, glomoid species found and richness assessed at Iturbe site of Puna.

ITURBE				
Trap Culture Strategy	It Field	ItM3-a1	ItM3-a2	ItM3-a3
Sampling site of Puna	Iturbe	Iturbe	Iturbe	Iturbe
Site elevation	3370 m	3370 m	3370 m	3370 m
Data source	[12]	This study	This study	This study
Initial total spore density/100 g dry soil weight	-	171	171	171
1st cycle time	-	5 months	5 months	5 months
1st cycle TPS	-	* Sorghum bicolor* *Melilotus albus* *Zea mays* *Pennisetum glaucum* *Avena sativa* *Secale cereale* * S. bicolor+ S. cereale * * P. glaucum+ S. cereale *	* S. bicolor* *M. albus* *Z. mays* *P. glaucum* *A. sativa* *S. cereale* *S. bicolor+ S. cereale* * P. glaucum+ S. cereale *	* S. bicolor* *M. albus* *Z. mays* *P. glaucum* *A. sativa* *S. cereale* *S. bicolor+ S. cereale* * P. glaucum+ S. cereale *
2nd cycle time	-	6 months	6 months	6 months
2nd cycle TPS	-	* S. bicolor *	* S. bicolor *	* S. bicolor *
3rd cycle time	-	2 months	2 months	2 months
3rd cycle TPS	-	* Allium ampeloprasum * var. *porrum*	* S. bicolor *	* M. albus *
Total time of trap culture		13 months	13 months	13 months
Glomoid AMF taxa	* Glomus * sp. *G. ambisporum* *Rhizoglomus aggregatus* (*= Glomus aggregatum*) *S. sinuosa* (*= Glomus sinuosum*)	* Funneliformis * sp. *F. geosporus* *F. monosporus* *R. aggregatus* *R. microaggregatum*	* Funneliformis * sp. *F. geosporus* *R. aggregatus* *S. sinuosa*	* F. geosporus* *R. microaggregatum* *R. aggregatus* *S. sinuosa *
Richness of glomoid morphospecies with funnel pore	0	2	2	1
Richness of total glomoid morphospecies	4	5	4	4
H’ Diversity Index (SSCP analysis)	-	1.46	1.52	1.54
Sequence of an excised band (SSCP analysis)	-	KT950814	KT950813	KT950812

It: Iturbe; It Field, Iturbe soil field samples; TPS: trap plant species; ItM3-a1: Iturbe soil samples with three cycles of trap culture with *Allium ampeloprasum* var. *porrum* as the third TPS; ItM3-a2: Iturbe soil samples with the three cycles of trap culture with *Sorghum bicolor* as the third TPS; ItM3-a3: Iturbe soil samples with three cycles of trap culture with *Melilotus albus* as the third TPS; SSCP: single-strand conformation polymorphism; -: no trap culture was carried out.

## Data Availability

The sequences data presented in this study are available in GenBank under the accession numbers (KT950811, KT950812, KT950813, KT950814, KT950815).

## Supplementary Materials

The following are available online at https://www.mdpi.com/article/10.3390/plants10091803/s1, Figure S1: Trap culture methodology applied to soil samples from the Puna Seca (Abra del Cóndor–Iturbe), Table S1: Environmental (climatic) characteristics of the Puna Seca (Abra del Cóndor–Iturbe) [25]. Table S2: Soil physicochemical characteristics at two elevations of the Puna [26,32]. Table S3: Dominant plant species sampled at two elevations of the Puna. Table S4: Results of BLASTn procedure and the criteria for selecting the homologous *Funneliformis* sequences used in the phylogenetic analysis.
