# Supplementary material for: Arbuscular Mycorrhizal Fungi from Argentinean Highland Puna Soils Unveiled by Propagule Multiplication
Source: Plants (Basel). 2021 Aug 30;10(9):1803. doi: 10.3390/plants10091803 (PMC8472016; doi:10.3390/plants10091803)
Supplement: Supplementary file 1 [file plants-10-01803-s001.zip › Figure S1.pdf]

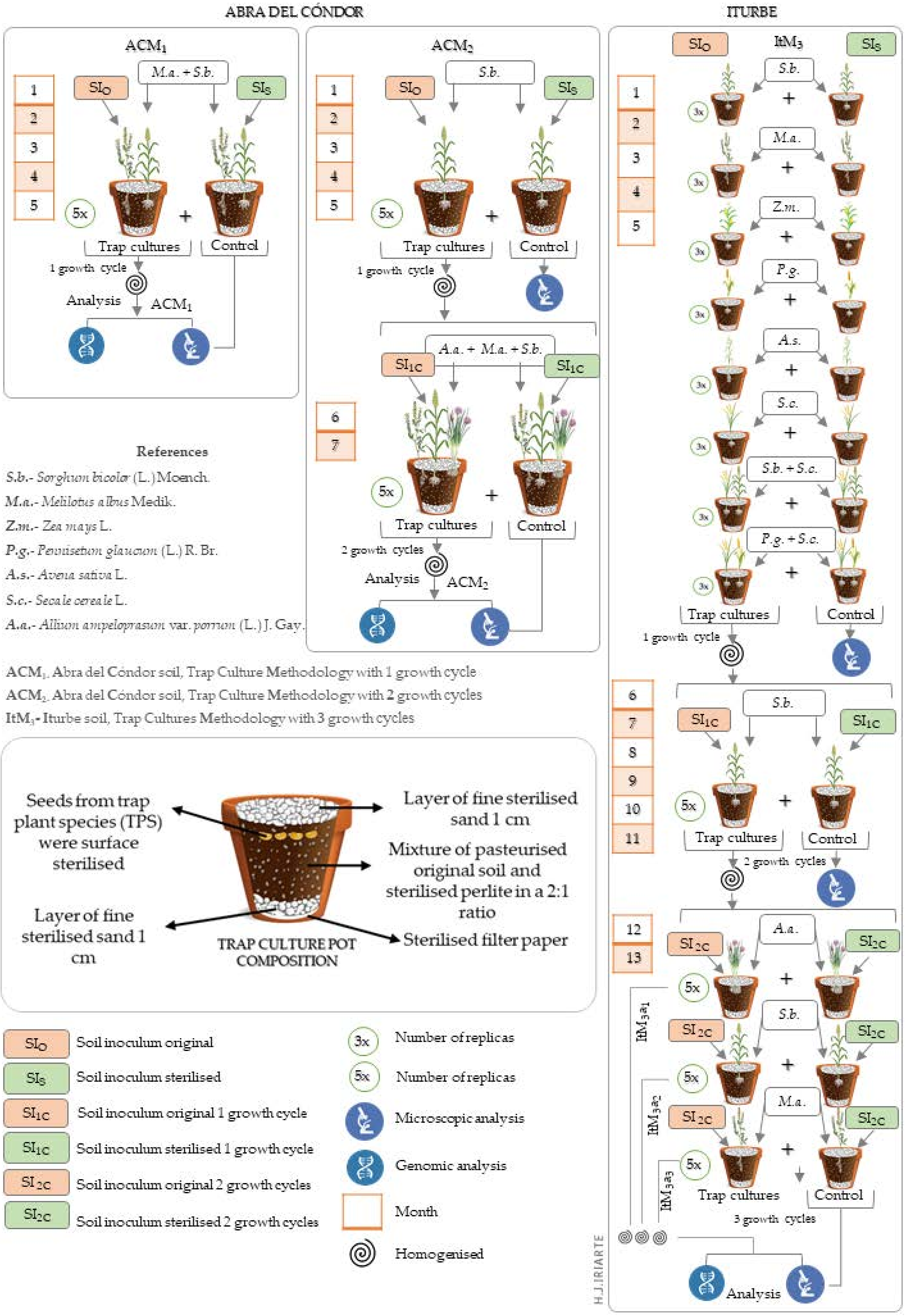

ABRA DEL CÓNDOR
ITURBE
ACM1
M.a. + S.b.
SIO
SIS
1
2
3
4
5
5x
Trap cultures
Control
1 growth cycle
Analysis
ACM1
ACM2
S.b.
A.a. + M.a. + S.b.
SI1C
6
7
2 growth cycles
ACM2
ItM3
M.a.
Z.m.
P.g.
A.s.
S.c.
S.b. + S.c.
P.g. + S.c.
3x
8
9
10
11
12
13
A.a.
SI2C
ItM3a1
ItM3a2
ItM3a3
3 growth cycles
References
S.b.- Sorghum bicolor (L.) Moench.
M.a.- Melilotus albus Medik.
Z.m.- Zea mays L.
P.g.- Pennisetum glaucum (L.) R. Br.
A.s.- Avena sativa L.
S.c.- Secale cereale L.
A.a.- Allium ampeloprasum var. porrum (L.) J. Gay.
ACM1, Abra del Cóndor soil, Trap Culture Methodology with 1 growth cycle
ACM2, Abra del Cóndor soil, Trap Culture Methodology with 2 growth cycles
ItM3- Iturbe soil, Trap Cultures Methodology with 3 growth cycles
Seeds from trap plant species (TPS) were surface sterilised
Layer of fine sterilised sand 1 cm
Mixture of pasteurised original soil and sterilised perlite in a 2:1 ratio
Layer of fine sterilised sand 1 cm
Sterilised filter paper
TRAP CULTURE POT COMPOSITION
SIO Soil inoculum original
SIS Soil inoculum sterilised
SI1C Soil inoculum original 1 growth cycle
SI1C Soil inoculum sterilised 1 growth cycle
SI2C Soil inoculum original 2 growth cycles
SI2C Soil inoculum sterilised 2 growth cycles
3x Number of replicas
5x Number of replicas
Microscopic analysis
Genomic analysis
Month
Homogenised
H.J.IRIARTE
