# Supplementary material for: Arbuscular Mycorrhizal Fungi from Argentinean Highland Puna Soils Unveiled by Propagule Multiplication
Source: Plants (Basel). 2021 Aug 30;10(9):1803. doi: 10.3390/plants10091803 (PMC8472016; doi:10.3390/plants10091803)
Supplement: Supplementary file 1 [file plants-10-01803-s001.zip › Table S1.pdf]

Table S1. Environmental (climatic) characteristics of the Puna Seca (Abra del Cóndor- Iturbe) [9]

Site	Precipitation			Solar radiation	Relative humidity	Daily thermal amplitude
	Mean annual	Winter (dry season)	Summer (rainy season)			
	-------------------mm---------------------			--UVI--	--%--	--ºC--
Puna Seca	340	50-70	300-500	>6	10-15	0-20
