# Supplementary material for: Arbuscular Mycorrhizal Fungi from Argentinean Highland Puna Soils Unveiled by Propagule Multiplication
Source: Plants (Basel). 2021 Aug 30;10(9):1803. doi: 10.3390/plants10091803 (PMC8472016; doi:10.3390/plants10091803)
Supplement: Supplementary file 1 [file plants-10-01803-s001.zip › Table S2.pdf]

Table S2. Soil physicochemical characteristics at two elevational sites of the Puna

Characteristics / Site	Abra del Cóndor (AC)	Iturbe (It)
Elevation (m)	3,870	3,370
Soil classification[1]	Entisol (torriontente lithic)	Aridisol (typical paleargid)
Texture	Gravel sandy	Sand silt gravel
pH	5.90± 0.03	6.80± 0.04
EC (mS/cm)	0.33 ± 0.01	0.33 ± 0.01
OM (%)	1.00 ± 0.30	1.00 ± 0.30
Cox.(%)	0.64± 0.02	0.50± 0.02
ON (g/Kg)	1.70 ± 0.10	0.60 ± 0.02
P^2 (ppm)	13.18± 0.86	34.30 ± 0.66
Ca^{2+} ($cmol_c$/Kg)	4.57 ± 0.28	3.36 ± 0.04
Mg^{2+} ($cmol_c$/Kg)	1.31 ± 0.11	0.69 ± 0.02
Na^+ ($cmol_c$/Kg)	0.43 ± 0.06	0.37 ± 0.03
K^+ ($cmol_c$/Kg)	0.95 ± 0.02	0.55 ± 0.02

[1][26]. CEC cation exchange capacity, EC electrical conductivity, OM organic matter, Cox. oxidable carbon, ON organic nitrogen, P^2 available soil phosphorus by Bray-Kurtz method [32]
