# Supplementary material for: Arbuscular Mycorrhizal Fungi from Argentinean Highland Puna Soils Unveiled by Propagule Multiplication
Source: Plants (Basel). 2021 Aug 30;10(9):1803. doi: 10.3390/plants10091803 (PMC8472016; doi:10.3390/plants10091803)
Supplement: Supplementary file 1 [file plants-10-01803-s001.zip › Table S3.pdf]

Table S3. Dominant plant species sampled at two elevational sites of the Puna

Site	Plant species
Abra del Cóndor	*Bouteloua barbata* (C_4), *Bromus catharticus* Vahl (C_3), *Danthonia annableae* P.M. Peterson & Rúgolo (C_3), *Jarava ichu* (Ruiz & Pav.) Kunth var. *pungens* (Nees & Meyen) Ciald. (C_3) and *Jarava subaristata* (Matthei) Matthei (C_3).
Iturbe	*Aristida asplundii* Henrard (C_4), *Bouteloua barbata* Lag. (C_4), *Cynodon dactylon* (L.) Pears. var. *biflorus* Merino (C_4), *Eragrostis nigricans* (Kunth) Steud. var. *punensis* Nicora (C_4), *Jarava plumosula* (Nees ex Steud) F. Rojas (C_3), and *Muhlembergia alopecuroides* (Griseb.) P.M. Peterson (C_4)
