# Supplementary material for: Arbuscular Mycorrhizal Fungi from Argentinean Highland Puna Soils Unveiled by Propagule Multiplication
Source: Plants (Basel). 2021 Aug 30;10(9):1803. doi: 10.3390/plants10091803 (PMC8472016; doi:10.3390/plants10091803)
Supplement: Supplementary file 1 [file plants-10-01803-s001.zip › Table S4.pdf]

Table S4. Results of Blastn procedure and the criteria for selection of homologous *Funneliformis* sequences used in phylogenetic analysis

	Sequences obtained in this study														
	KT950811			KT950812			KT950813			KT950814			KT950815		
**Sequences available at NCBI and/or MAARJAM databases**	E-value	Query cover* %	Max ident* %	E-value	Query cover* %	Max ident* %	E-value	Query cover* %	Max ident* %	E-value	Query cover* %	Max ident* %	E-value	Query cover* %	Max ident* %
Funneliformis mosseae (JQ048902)	5e-152	100	99.34	7e-132	100	98.5	2e-161	99.7	99	2.4e-154	99.7	97.7	2.8e-161	100	99
F. mosseae (GU966531)	5e-152	100	99.34	7e-125	100	98.5	-	-	-	-	-	-	5e-152	100	99.34
F. mosseae (JF439112)	1e-152	99	99.7	-	-	-	-	-	-	1e-143	99	97.7	2.8e-161	100	99
F. mosseae (FR750030)	1e-152	99	99.7	-	-	-	1.5e-159	99.7	98.7	1.3e-152	99.7	97.4	2.8e-161	100	99
F. mosseae (FN547490)	1e-152	99	99.7	-	-	-	1.5e-159	99.7	98.7	1.3e-152	99.7	97.4	1e-152	99	99.7
F. mosseae (AF145735)	1e-162	99.7	99.3	-	-	-	-	-	-	-	-	-	-	-	-
F. caledononium (MH590067)	2e-141	99	96.3	1e-118	100	95.7	2e-141	99	96	2e-134	99	94.3	4e-144	100	96
F geosporum (JQ048887)	2e-141	99	96.3	1e-117	100	95.4	1e-132	99	94	2e-128	99	93	2e-136	100	95
F. coronatum (FM876795)	3e-139	99	96	7e-115	100	95	1e-138	99	95.7	4e-132	99	94	3e-140	100	96

Septoglomus desertícola (AJ746249)	4e-112	99	89.7	1e-92	100	88.5	5e-111	99	88.7	2e-111	99	88.7	8e-115	100	89.7
Scutellospora calospora (JF439140)	2e-64	66	93.7	4e-61	62	92	9e-64	67	92.6	7e-65	53	93	7e-65	67	93.2
Ambispora gerdemanni (KC166283)	1e-62	50	93.5	1e-58	60	91.1	2e-60	51	91.7	4e-62	51	92.4	4e-62	52	92.4
Acaulospra lacunosa (KP756582)	4e-61	46	96.4	2e-58	54	94.4	2e-60	47	95	4e-62	47	95.8	2e-62	47	95.8
Gigaspora margarita (JF817010)	1e-62	76	93.5	2e-59	61	91.8	2e-60	52	91.8	3e-63	52	93.1	3e-63	77	93.1

* Query coverage, Max. identity
